# Microglia and Brain Plasticity in Acute Psychosis and Schizophrenia Illness Course: A Meta-Review

**DOI:** 10.3389/fpsyt.2017.00238

**Published:** 2017-11-16

**Authors:** Livia J. De Picker, Manuel Morrens, Steven A. Chance, Delphine Boche

**Affiliations:** ^1^Collaborative Antwerp Psychiatric Research Institute, University of Antwerp, Antwerp, Belgium; ^2^University Psychiatric Center St. Norbertus, Duffel, Belgium; ^3^Nuffield Department of Clinical Neurosciences, West Wing, John Radcliffe Hospital, University of Oxford, Oxford, United Kingdom; ^4^Clinical Neurosciences, Clinical and Experimental Sciences Academic Unit, Faculty of Medicine, University of Southampton, Southampton, United Kingdom

**Keywords:** schizophrenia, psychosis, microglia, neuroinflammation, neuroplasticity, translocator protein, positron emission tomography, *post-mortem* study

## Abstract

**Objective:**

Schizophrenia poses a tremendous health, social, and economic burden upon patients and society, indicating current treatment options remain inadequate. Recent findings from several lines of evidence have pointed to the importance of immune system involvement in not only premorbid neurodevelopmental but also subsequent symptom generation and aging processes of brain change in schizophrenia. In this meta-review, we use the summarized evidence from recent quantitative systematic reviews (SRs) and meta-analyses of several subspecialties to critically evaluate the hypothesis that immune-related processes shape the symptomatic presentation and illness course of schizophrenia, both directly and indirectly through altered neuroplasticity.

**Methods:**

We performed a data search in PubMed for English language SRs and meta-analyses from 2010 to 2017. The methodological quality of the SRs was assessed with the AMSTAR instrument. In addition, we review in this paper 11 original publications on translocator protein (TSPO) positron emission tomography (PET) imaging in schizophrenia.

**Results:**

We reviewed 26 SRs and meta-analyses. Evidence from clinical observational studies of inflammatory or immunological markers and randomized controlled drug trials of immunomodulatory compounds as add-on in the treatment of schizophrenia suggests psychotic exacerbations are accompanied by immunological changes different from those seen in non-acute states, and that the symptoms of schizophrenia can be modified by compounds such as non-steroidal anti-inflammatory drug and minocycline. Information derived from *post-mortem* brain tissue analysis and PET neuroimaging studies to evaluate microglial activation have added new perspectives to the available evidence, yet these results are very heterogeneous. Each research domain comes with unique opportunities as well as inherent limitations. A better understanding of the (patho-)physiology of microglial cells and their role in neuroplasticity is key to interpreting the immune-related findings in the context of schizophrenia illness exacerbations and progression.

**Conclusion:**

Evidence from clinical studies analyzing patients’ blood and cerebrospinal fluid samples, neuroimaging and *post-mortem* brain tissue suggests that aberrant immune responses may define schizophrenia illness’ course through altered neuroplasticity representing abnormal aging processes. Most findings are however prone to bias and confounding, and often non-specific to schizophrenia, and a multidisciplinary translational approach is needed to consolidate these findings and link them to other schizophrenia hypotheses.

## Introduction

Schizophrenia is a devastating and complex central nervous system (CNS) disorder that affects approximately 1% of the world population. Despite antipsychotic drugs being available for several decades, personal and societal costs remain highly expensive: the World Health Organization estimated that the direct costs of schizophrenia range between 7 and 12% of the gross national product in Western countries (1). Current treatment options are inadequate, both due to side-effects that disable patients and threaten their compliance to treatment (2) as well as the drugs’ lack of efficacy evidenced by the absence of clinically relevant improvements on negative and cognitive symptoms, and up to one-third of schizophrenia patients not responding at all (3–5). Acute psychosis is common and relapse prevention represents an important treatment issue in schizophrenia. An estimated 82% of patients have an illness relapse within 5 years after recovery from first-episode psychosis (FEP), and a majority will experience multiple relapses throughout their illness course (6). Furthermore, a high relapse rate is associated with poorer outcomes, including more treatment-resistant symptoms, cognitive deficits, and functional disability (7, 8).

These problems emphasize the urgent need for novel therapeutic approaches implicating the identification of new pathophysiological substrates. The illness is syndromic, of heterogeneous presentation and course and while there are well-established criteria in place for making the diagnosis of schizophrenia, the causes are still under debate. This is partly due to the complexity of this human disease that cannot be wholly replicated in animal models (9), as well as the methodological difficulties related to studying brain functionality in living patients. With increased understanding of heritability and ongoing brain changes, a vulnerability–stress model (sometimes described as a two-hit model) has become a prominent hypothesis to explain the pathophysiology of schizophrenia, in which an interaction of genetic, epigenetic, and environmental risk factors is assumed to result in brain abnormalities that continue to generate abnormal responses throughout life upon exposure to relevant stressors (10). This presents a modification of the original neurodevelopmental hypothesis which proposed that “a fixed lesion early in life interacts with normal brain maturational events that occur much later” (11). Indeed, it now appears that the neurodevelopmental concept should be extended to encompass altered ongoing neuroplasticity (12–14) creating a background of susceptibility for new insults later in life to catalyze neurodegenerative effects.

In the late twentieth century, the investigation of astrogliosis as hallmark for an inflammatory reaction in the schizophrenic brain combined with the expansion of neuropsychiatric genetics shape the debate about the etiology of schizophrenia. The absence of neuroinflammation and the presence of heritable candidate genes were thought to indicate a neurodevelopmental origin and a static rather than a neurodegenerative disease course. Although the earliest study reported the presence of low-grade inflammation (15), subsequent efforts did not replicate this finding. Consequently, the consensus was that reactive astrogliosis is absent in schizophrenia. However, the refinements of immunohistochemistry and modern stereological microscopy have yielded evidence for glial changes and, at the same time, brain imaging studies have increasingly demonstrated that the schizophrenic brain is not static during its clinical course. Over the last decades, immune system involvement has increasingly been implicated in the pathophysiological processes in schizophrenia (16–18). It has been hypothesized to be a driving factor behind both psychotic relapses and the macroscopic brain changes that occur in schizophrenia, including the characteristic enlarged ventricle size and reductions in gray matter volume, whole-brain volume, and white matter anisotropy (14).

The field has now advanced enough for several major meta-analyses and systematic reviews (SRs) to have come out in recent years, each compiling the evidence derived from individual studies and large groups of subjects in a variety of sub-disciplines contributing to this work. This meta-review aims to condense the evidence that immunological factors are critically involved in the schizophrenia illness course—modifying symptomatology and prognosis due to progressive brain changes. We critically appraise the current literature, identifying the opportunities and methodological limitations arising from each of the different lines of evidence. We identify areas where crucial information is still lacking and defend the position that opportunities can emerge by adopting a multidisciplinary translational approach to consolidate current findings and link this field of research to other key hypotheses of schizophrenia research. As the schizophrenia psychoneuroimmunology literature is very large, the scope of this review is necessarily limited. The focus will be on specific topics in the schizophrenia literature on neuroinflammation: (1) *in vivo* neuroimaging and (2) neuropathological studies of microglial activation; (3) systemic inflammation and its link to psychosis; (4) clinical trials of immunomodulatory drugs as add-on treatment; and (5) neuroplasticity. Other relevant domains such as epidemiological studies, genetic studies, animal studies and clinical studies on changes in related mechanisms such as the kynurenine pathway and oxidative stress markers have their own sub-literature, and will not be dealt with here.

## Methods

We undertook a qualitative “review of reviews” outlining the summarized evidence for immune system involvement in the illness course of schizophrenia. The PubMed database was searched for English-language publications from January 2010 to August 2017. The different search strings employed per domain are detailed below. For each of these, the PubMed filter “Article Type” was set to categories “Systematic Review” and “Meta-analysis.” Besides the articles found through the PubMed database, a manual review of reference lists of included articles was performed to identify additional papers. A consensus was reached among authors on the studies retained or discarded on the basis of the following inclusion and exclusion criteria. The included articles were (1) meta-analyses and systematic quantitative reviews; (2) investigating immune-related alterations in the blood, cerebrospinal fluid (CSF), or brain (3) of patients meeting DSM-IV or DSM-V criteria for the schizophrenia spectrum. All included studies are summarized in Table 1. We excluded publications focusing on preclinical research, or premorbid immunological risk factors such as epidemiological or genetic background risks. Articles were excluded if they were: (1) narrative or qualitative review articles; (2) reviews on topics outside the scope of this paper (as indicated in the inclusion criteria); (3) reviews of *in vitro* or animal studies; (4) reviews of genome or gene expression studies; (5) reviews of epidemiological risk factors for schizophrenia; or (6) not published in English. We assessed the methodological quality of the SRs using the AMSTAR instrument (19); scores are presented in Table 1.

**Table 1:** Summarized evidence of immune processes influencing the course of schizophrenia.

	Research methods	MA/SR	*N* studies	*N* patients	AMSTAR score (out of 11)	Outcomes
Clinical studies on peripheral expression of biomarkers and cells	Case–control study on blood and CSF inflammatory markers and immune cells (patients versus controls OR *post-mortem* comparison of patients in different states)	Miller (20): MA blood cytokine studies	40	1,324 SZ; 1,154 C	9	Trait and state increases of inflammatory cytokines; in schizophrenia patients, blood *trait* markers are IL12, IFNγ, TNFα, sIL2R, CRP, CD56 cells; *state* markers are IL1β, IL6, TGFβ, CD4 cells; in unmedicated FEP blood trait markers are TNFα, IL17, and IFNγ, state markers are IL6 and IL2, and possibly IL1β; in CSF increased IL1β, IL6, IL8

		Miller (21): MA blood lymphocyte studies	16	488 SZ; 525 C	9	

		Tourjman (22): MA blood cytokine studies and AP	23	762 SZ	6	

		Miller (23): MA blood CRP studies	8	N/A	N/A	

		Upthegrove (24): MA blood cytokine studies in drug-naïve FEP	23	570 SZ; 683 C	9	

		Guo (25): MA blood cytokine studies	21	N/A	N/A	

		Goldsmith (26): MA blood cytokine studies acute–chronically ill	40	N/A	N/A	

		Fernandes (27): MA blood CRP studies	71	85,000 SZ + C	N/A	

		Wang and Miller (28): MA CSF cytokine studies	16	N/A	N/A	

		Capuzzi (29): MA blood cytokine studies on effect of AP in FEP drug-naive patients	8	505 SZ	6	

	Case–control study on autoantibody titers	Ezeoke (30): MA antibody titer studies	81	N/A	8	Increased prevalence of positive titers for 20 different autoantibodies

		Monroe (31): MA *T. gondii* titer studies	16	2,535 SZ; 1,707 C	9	Increase in *T. gondii* IgM antibodies in acute psychosis

*Post-mortem* studies	Case-control study on histological or molecular findings in *post-mortem* brain tissue	Bakhshi (14): SR of neuronal density studies	30	360 SZ; 390 C	4	Age-dependent increases in neuronal density

		Najjar and Pearlman (32): SR white matter pathology and neuroinflammation studies	15	350 SZ; 346 C	5	Approximately half of *post-mortem* studies reported increased microglial markers

		Trepanier (33): SR neuroinflammation markers	119	N/A	4^a^	Similar patterns of heightened innate immune gene expression in both brain and blood in schizophrenia

		Hess (34): 2 mega-analyses of microarray data from *post-mortem* prefrontal cortices and *ex vivo* blood tissues	19 (brain)	315 (brain)	N/A	
			25 (blood)	578 (blood)		

		Van Kesteren (35): MA histological/molecular immunological parameter studies	41	783 SZ; 762 C	9^a^	

Neuroimaging	Case–control study on PET imaging for microglial activation	Data summarized in Table 2	11	176 SZ; 175 C	N/A	Increased uptake of tracer with certain tracers and kinetic models in certain patient populations; results vary

Clinical trials	RCT with adjunctive anti-inflammatory drugs	Sommer (36): MA RCT NSAID	5	264 SZ	N/A	Results vary, significant effects have been found for aspirin, celecoxib, estrogens, NAC, and minocycline

		Nitta (37): MA RCT NSAID	8	774 SZ	10	

		Sommer et al. (38): MA RCT anti-inflammatory agents	24	1,938 SZ	8	

		Oya (39): MA RCT minocycline	4	330 SZ	10	

		Solmi (40): MA RCT minocycline	6	413 SZ	11	

		Xiang (41): MA RCT minocycline	8	548 SZ	9	

		Zheng (42): MA RCT celecoxib	8	626 SZ	9	

*SZ, schizophrenia patient group; C, control group; n, number of subjects; FEP, first-episode psychosis patients; MA, meta-analysis; SR, systematic review; N/A, not applicable/not available; ES, effect size; RCT, randomized controlled trial; RR, relative risk; CSF, cerebrospinal fluid; PET, positron emission tomography; NSAID, non-steroidal anti-inflammatory drug; NAC, N-acetylcysteine*.

*^a^Included results from a paper which was later retracted*.

Meta-analyses of immunological or inflammatory changes in the blood, CSF and brain of schizophrenia patients were identified using the following search string: schizophrenia[Title/Abstract] AND (*inflamm*[Title/Abstract] OR *immune*[Title/Abstract] OR cytokine*[Title/Abstract] OR chemokine*[Title/Abstract] OR *glia*[Title/Abstract]). 82 articles were retrieved, of which 15 were included. Other studies were excluded because they did not study schizophrenia patients (*n* = 15); concerned genome or gene expression studies (*n* = 11); were narrative or qualitative review papers (*n* = 13); or were outside the scope of this paper (*n* = 24). Two additional articles were added manually after review of the reference list.

To identify meta-analyses pertaining to randomized controlled trials (RCTs) of anti-inflammatory agents in schizophrenia, the following search string was used: schizophrenia[Title/Abstract] AND (RCT*[Title/Abstract] OR randomized[Title/Abstract]) AND (minocycline[Title/Abstract] OR NSAID[Title/Abstract] OR *inflamm*[Title/Abstract]). This generated 11 articles, of which 8 were included. Three studies were excluded for the following reasons: does not concern clinical trials (*n* = 1); is a narrative review (*n* = 1); compound is outside the scope of paper (*n* = 1).

Because no meta-analysis or SR could be identified on positron emission tomography (PET) studies of microglial activation in schizophrenia, we chose to add the eleven original studies to this paper, as this is an important sub-discipline in this research field. These papers were identified using a Pubmed and Medline search executed in August 2017 without limitation of time, language, or publication type. The search string combined following terms: (schizophrenia[Title/Abstract] OR psychosis[Title/Abstract]) AND (TSPO*[Title/Abstract] OR Translocator[Title/Abstract] OR *PK11195[Title/Abstract] OR *PBR*[Title/Abstract]).

## Microglial Activation Influencing the Course of Schizophrenia

The hallmark of neuroinflammation is the activation of microglia, the resident immune cells in the CNS (43), which are central to the current immune-related hypothesis in schizophrenia. Microglia are critically involved in the organization of the neuronal network during brain development (44) by primarily pruning excess synapses (45). Experimental models of schizophrenia showed that maternal infection during embryogenesis contributes to the presence of activated microglia in the offspring (46). This suggests that neonatal infections might induce perinatal microglial priming (47), a process in which highly sensitized microglia lead to an exaggerated response by example during the maturation processes occurring at the adolescent age and which can result in behavioral changes (48–50). This has led to the so called “microglia hypothesis” of schizophrenia, positioning exaggerated microglial activation as a key factor in the etiology of schizophrenia (17). In this hypothesis, exposure to inflammatory responses and/or genetically rooted excessive synapse pruning in the perinatal period (51) triggers an activated or primed state of microglia in adulthood (17), leading to ongoing systemic and central abnormal immune responses which drive schizophrenia symptoms such as psychotic episodes, and cause an unfavorable illness course, with progressive loss of function.

The presence of such ongoing microglial activation in schizophrenia patients has been supported both by some *post-mortem* studies as well as a few *in vivo* PET imaging studies using specific ligands (32, 52) that visualize and quantify microglial activation.

### Neuroimaging Studies on Microglial Activation

PET radioligands for microglial activation have recently been developed allowing for the first time functional imaging of neuroinflammation *in vivo*. These radiotracers selectively bind to the 18-kDa translocator protein [translocator protein (TSPO), previously called peripheral benzodiazepine receptor], which is expressed on the outer mitochondrial membrane of microglia in their activated states. Interestingly, although the (patho-)physiological function of the TSPO, which is related to cholesterol transport for steroid production in microglia, is not well understood (53), these tracers have been proven very useful and are now increasingly being used for the prospective *in vivo* study of human microglial activation in several CNS conditions. This means the evolution of early and late inflammatory changes can be followed over months and correlated with symptom severity in clinical longitudinal studies. The technique also allows to monitor the effects of treatment and to evaluate new anti-inflammatory or neuroprotective strategies. The oldest and most frequently used tracer is PK11195 radiolabeled with carbon-11, but its high lipophilicity and non-specific binding are thought to impede accurate quantification of tracer uptake. Since then, several newer second-generation tracers have emerged for use with either carbon-11 or fluor-18 labeling, boasting an improved pharmacokinetic profile and an up to 80-fold higher specific binding as demonstrated by animal blocking studies (54). Second-generation tracers radiolabeled with 18-F, which has a longer half-life, have made this technique available for nuclear imaging centers without cyclotron, although their use is complicated by the existence of a polymorphism (*rs6971*) in humans that occurs in 30% of the European population. Therefore, genotyping of study subjects at this locus is essential to allow interpretation of TSPO PET studies, by stratifying between genetic groups and exclusion of low-affinity subjects.

As a meta-analysis is lacking for this specific research domain, the results of the existing studies are summarized in Table 2. Interestingly, whereas some PET studies have revealed that activated microglia are present in frontal and temporal lobes and total gray matter in the following: participants at ultra-high risk of psychosis (55), patients within the first 5 years of disease onset (56) or during a psychotic state (57); other PET studies have shown no difference in microglial activation between healthy controls and patients in various clinical states (ultra-high risk, first-episode psychosis (FEP), after a recent diagnosis of schizophrenia or in chronic phases of the illness) (58–63); or even decreased uptake in first-episode medication-naïve patients (64). With four new negative papers published in the last year (61–63, 65), it seems this field is experiencing an episode of catharsis after the initial enthusiasm that accompanied the first positive exploratory results almost ten years earlier, and it is becoming increasingly unclear whether there is indeed enhanced TSPO uptake in schizophrenia.

**Table 2:** Positron emission tomography studies with TSPO tracer evaluating microglial activation in schizophrenia patients *versus* controls.

Reference	*n* SZ	*n* C	Tracer	Model; outcome measure	Clinical state	DOI (years)	Medication	Outcome
					Total (T) and positive symptom scale (P) score (PANSS mean ± SD unless otherwise specified)		% of patients on antipsychotics (AP); (mean CPZ) Benzodiazepines (BZD) excluded (duration)?	
Van Berckel *et al*. (56)	10	10	[11C]PK11195	2TCM; BP	Undefined	3.1 ± 1.7	AP 100%	SZ **>** C
					Symptom scores unavailable		BZD?	

Doorduin *et al*. (57)	7	8	[11C]PK11195	2TCM; BP	Psychosis	5 ± 6	AP 100%	SZ **>** C
					T 73.6 ± 13.3		BZD excluded (3 × *t*_1/2_)	
					*P* 19.7 ± 3.0			

Banati and Hickie (66)	16	8	[11C]PK11195	2TCM; BP	Undefined	Range 0.3–30	Information unavailable	SZ **>** C
					Symptom scores unavailable			

Takano et al. (58)	14	14	[11C]DAA1106	2TCM; BP	Chronic	19 ± 12	AP 100%	SZ **=** C
					T 77.9 ± 20.1		BZD excluded (<1 m)	
					*P* 19.1 ± 5.3			

Kenk et al. (59)	16	27	[18F]FEPPA	2TCM; *V*_T_	Psychosis	15 ± 9	AP 100%; (300 CPZ)	SZ **=** C
					T 70.2 ± 9.7			
					*P* 19.3 ± 2.2		BZD excluded (duration?)	

Bloomfield et al. (55)	14	14	[11C]PBR28	2TCM-1K; DVR	Undefined	Undefined	AP?%	SZ **>** C
					T 63.7 ± 18.1		BZD excluded (duration?)	
					*P* 17.0 ± 6.1			

Coughlin et al. (60)	12	14	[11C]DPA713	Undefined; *V*_T_	Undefined	2.2 ± 1.4	AP?%; (474.5 CPZ)	SZ **=** C
					T unavailable			
					*P* (SAPS) 3.8 ± 2.5		BZD excluded (6 m)	

Van der Doef et al. (61)	19	17	[11C]PK11195	Reference tissue; BP	Undefined	1.3 ± 1.1	AP 79%	SZ **=** C
					T 53 ± 10		BZD excluded (4w)	
					*P* 12 ± 4			

Collste et al. (64)	16	16	[11C]PBR28	2TCM; *V*_T_	FEP drug naïve	0.7 ± 0.8	AP 0%	SZ **<** C
					T 77.4 ± 18.3		BZD not excluded	
					*P* 20.3 ± 4.9			

Hafizi et al. (65)	19	20	[18F]FEPPA	2TCM; *V*_T_	FEP unmedicated	2.8 ± 3.3	AP 0%	SZ **=** C
					T 68.6 ± 13.0		BZD?	
					*P* 19.2 ± 3.8			

Di Biase et al. (62)	33	27	[11C]PK11195	Reference tissue; BP	Recent-onset (*n* = 18) T 68.5^a^ *P* (BPRS) 12.6 ± 4.6	1.5 ± 1.0	AP 78% BZD?	SZ = C
					Chronic (*n* = 15) T 86.5^a^ *P* (BPRS) 19.5 ± 7.8	13.6 ± 8.8	AP 100% BZD?	SZ = C

*SZ, schizophrenia patient group; C, control group; *n*, number of subjects; FEP, first-episode psychosis patients; DOI, duration of illness; 2TCM, two-tissue compartment model; BP, binding potential; *V*_T_, total volume of distribution; DVR, distribution volume ratio; CPZ, chlorpromazine equivalent; SAPS, Scale for the Assessment of Positive Symptoms; ?, undefined*.

*SZ > C, increased uptake of tracer in schizophrenia patients compared to controls*.

*SZ = C, no difference in tracer uptake between schizophrenia patients and controls*.

*SZ < C, decreased uptake of tracer in schizophrenia patients compared to controls*.

*^a^Mean Brief Psychiatric Rating Scale total scores were converted to corresponding Positive and Negative Syndrome Scale total scores using the equipercentile linking method (67)*.

One major drawback of this new technology is that it comes with considerable technical requirements. First, the kinetic properties of the TSPO tracers currently require complex dynamic scanning protocols (60–125 min PET scan, often with an arterial line) which are considered a challenging and relatively invasive procedure toward patients. Second, because of the low resolution yielded by PET, co-localization with additional magnetic resonance imaging (MRI) images is essential to assess regional specific uptake. Taken together with the restriction for patients not to use benzodiazepines prior to the scan, these technical requirements arguably compromise the generalizability of PET study samples due to significant non-response bias in patient groups with the highest symptomatic burden and limit the future clinical use of the procedure.

For studies to be conducted effectively, adequate statistical power is necessary, yet because of the high technical and financial burden of these studies, sample sizes are typically rather small. Even after stratifying for genotype, high inter-subject variability is seen in imaging with TSPO tracers. This variability may be due to technical variables such as (i) the degree of tracer plasma protein binding, which is estimated to introduce up to 30% of variability in primary outcome measure *V*_T_ (55), (ii) a relatively low signal-to-noise ratio (particularly with first-generation tracers), or (iii) other unidentified technical or clinical confounders. Intra-subject variability, measured as test–retest reliability, depends upon a number of factors including scanner noise, input function noise, tracer kinetics, and any fluctuating biological variability of the target within the inter scan period, with decreased reliability causing a loss of statistical power that is not accounted for in typical sample size calculations. For second-generation TSPO tracers, intra-class correlation coefficients have been reported around 0.76–0.92 for gray matter and 0.32–0.64 for white matter (64) (Ottoy et al., submitted).

Furthermore, a complicated kinetic modeling procedure is involved, in which the kinetic properties of the tracer together with the chosen mathematical model and outcome measure have a large impact on the results, as evidenced by the inconsistent results found across the TSPO PET imaging studies. The dominant methodology has been to adopt a regular two-tissue compartmental model (2TCM). However, a model which also accounts for endothelial and vascular TSPO binding (2TCM-1K) was recently shown to have improved performance in second-generation TSPO tracers, probably because in these tracers a higher ligand specific TSPO affinity leads to considerable endothelial binding in the blood–brain barrier. Additionally, a less invasive reference tissue model was developed to study TSPO binding using first-generation tracer PK11195 without arterial input function (61). A second important consideration is that the outcome measure is normally either BP (binding potential, i.e., a combined measure of the density of available receptors and the affinity of the radioligand to that receptor) or *V*_T_ (total volume of distribution, i.e., the ratio of the radioligand concentration in the region of interest to that in plasma at equilibrium). This means the increased uptake observed in a certain region of interest could reflect either increased non-specific tracer binding as well as a true biological signal (68). Strikingly in one of the studies, no difference was found between patients and controls when using the regular outcome measure *V*_T_; yet when the authors accounted for inter-subject variability in the input function by using DVR (distribution volume ratio, i.e., the ratio of the *V*_T_ in the region of interest to *V*_T_ in the whole brain) as their outcome measure, large effect sizes (Cohen’s *d* > 1.7) were found (55). This alternative region-based approach has been subject to the criticism that it does not clarify whether the higher distribution volume ratio values in schizophrenia subjects are a result of greater microglial activation in the region of interest or of lesser microglial activation in other regions included in the denominator of the outcome measure, such as the cerebellum or white matter (69). The authors of the original study thereupon defended their choice by explaining DVR is superior to *V*_T_ when plasma input function measures are unreliable (such as is the case with TSPO tracers, which generally have <5% free fraction in plasma) or subject to systematic group differences, for instance caused by elevated acute phase plasma proteins and cytokines in schizophrenia (70).

Also remarkably, when one of the pioneering author groups in this field replicated their study in a larger patient sample and using a reference region instead of arterial input function, they no longer found a difference between patients and controls as they did in the original study (56, 61).

Finally, the direct biological relationship between microglia and TSPO binding *in vivo* is still not fully understood. The exact cellular source of the upregulated TSPO binding in CNS pathology remains subject to discrepancy, caused by the extrapolation of *in vitro* data to the *in vivo* situation and differences in the models used (for instance lesions with or without blood–brain barrier damage) with literature reporting TSPO expression not only on microglial cells but also on astrocytes and some neuronal subtypes, as well as endothelial cells. In non-human primates, endotoxin-induced systemic inflammation caused marked increases in the signal of a second-generation TSPO tracer, confirmed *post-mortem* to be largely due to microglial binding (71). Although this animal model is not suitable to study the brain changes of schizophrenia patients—which are much more subtle than those in many “typical” inflammatory disorders and autoimmune disorders—the source of TSPO radioligand binding has not yet been studied in more relevant models such as the (maternal) Poly I:C viral mimetic challenge. Given the subtlety of these changes as well as the different cell types involved, it is possible to imagine a scenario in which small increases and decreases in different cell types could cancel each other out causing no diagnosis-related differences regarding TSPO binding to be found despite biological changes being present.

Finally, even if increased TSPO binding truly reflects increased microglial activation, PET images do not provide the detailed morphological and functional data required to appreciate the function of microglia in pathophysiological states. Interpretation of such findings is therefore complex as they may reflect both a protective response and a neuroinflammatory process which causes psychotic symptoms and/or neurodegenerative effects.

### Neuropathological Studies on Microglia

A recent paper of two mega-analyses of combined micro-array data in brain and blood tissues re-emphasizes the raised expression of innate immune system genes in the brain but also in the blood of people with schizophrenia (34). However, anatomopathological investigation has been the gold standard for thorough characterization and investigation of human tissue. It offers the best possible resolution to evaluate changes at the cell level as well as interactions between microglial cells and other brain functional components such as neurons. Although data derived from *post-mortem* brain tissue of schizophrenia patients are scarce, an association between schizophrenia and microglial activation, particularly in white matter regions, has been observed (49). Yet a definitive statement cannot be made on whether neuroinflammation is present in schizophrenic *post-mortem* brain samples due to the significant number of negative studies and conflicting results that have been published by different groups with sometimes the use of the same microglial marker (59).

As the largest meta-analysis in this field, Trepanier et al. reviewed a total of 119 articles on neuroinflammation in *postmortem* schizophrenic brains, 22 of which looked at a range of microglial markers. Of these, 11 studies reported an increase in microglial markers in *post-mortem* brains, whereas 8 studies found no effect and 3 studies found a decrease in microglial markers. HLA-DR is the strongest risk factor for schizophrenia and a component of the major histocompatibility class II involved in “non-self” recognition, antigen presentation and the most used marker of microglial activation in schizophrenia. The SR by Trepanier et al. reported differential expression of HLA-DR by immunohistochemistry in 11 out of 13 *post-mortem* studies (9 studies reporting increased and 2 decreased expression). The same review reported unchanged expression in 3 out of 3 studies using CD68, a marker of microglial lysosomes indicative of phagocytic microglia and involved in clearance of neuronal debris in neurodegeneration, and 2 out of 2 studies with Iba1 (ionized calcium-binding adapter molecule 1), a protein involved in membrane ruffling (72) and thus a marker associated with microglial motility, a function essential to brain surveillance (73).

More recently, Van Kesteren *et al*. reviewed 41 studies on immune involvement in *post-mortem* schizophrenic brains, with 11 studies investigating microglia on 181 patients and 159 controls. A significant increase in microglia was observed [standard mean deviation (SMD) = 0.69**] over all studies, yet again with significant heterogeneity. The authors also reported a significant increase in the overall expression of pro-inflammatory molecular components (14 studies on 330 patients and 323 controls; SMD = 0.37**).

It is worth noting that these two main meta-analyses included among their results a paper which demonstrated increased microglial staining and increases in IL1β and TNF expression in the frontal cortex, but was retracted afterward (74).

The most evident problem for this kind of study is the limited availability of patients’ brain tissue. Since *post-mortem* research is by definition at the end of life, many clinical confounders related to the life and illness history of both patients and controls may complicate the interpretation of data. Known confounders include gender, age at death, use of medication, etc. The cause of death should also be considered, as the presence of systemic inflammation, hematological malignancies or neurological illness may confound the results, as can suicide, which is common in schizophrenia and has been linked to the presence of elevated pro-inflammatory cytokines regardless of diagnosis (75). It is therefore essential that all tissue samples are accompanied by detailed clinical information and that the control group has been mindfully selected. Heterogeneity may also be explained in part by the qualitative vs. quantitative analysis, the brain region and cortical layer in which the markers are measured (59). Furthermore, there may be differences in the diagnosis methods, inclusion and exclusion criteria, storage conditions, and demographics variables among the brain banks which provide the brain samples.

Besides the microglial cells, structural and functional abnormalities in the other two glial cell types have also been studied in relation to inflammation in schizophrenia. For instance, evidence related to reduced numbers and impaired cell maturation of oligodendrocytes has been linked to white matter abnormalities and disturbed inter- and intra-hemispheric connectivity. Astrocyte dysfunction, as suggested by some studies demonstrating abnormal expression of a variety of astrocyte-related genes as well as S100B protein, may contribute to certain aspects of disturbed neurotransmission in schizophrenia (76). However, these studies fall outside the scope of this paper and are not reviewed here.

## Systemic Inflammation and Its Association with Psychosis in Schizophrenia

Clinical research has focused to a great extent on peripheral immune system alterations in adult schizophrenia patients. Clinical observational studies in adult patients are relatively easy to organize and considered non-invasive, as blood sampling is often part of routine medical practice. The resulting large sample sizes and a lower selection bias imply study samples are generally more representative of clinical populations. Furthermore, the identification of potential peripheral biomarkers will be useful to confirm diagnostic or prognostic questions in clinical practice.

To assess the role of immunity in illness symptomatology, it is important to carefully differentiate patient groups according to their clinical characteristics. This was done for the first time by Miller *et al*., who reviewed published data of levels of cytokines (or cytokine receptors or antagonists), and later also of blood monocytes or lymphocytes, *Toxoplasma gondii* and auto-antibody titers in patients with schizophrenia or related psychotic disorder and healthy control subjects in a series of meta-analyses. Their analyses included both cross-sectional studies and longitudinal studies in patients with an acute exacerbation of psychosis at baseline and following a period of antipsychotic treatment. All studies included in this paper met strict criteria for defining patients’ clinical status [as acutely relapsed inpatients (AR), FEP, stable medicated outpatients or treatment-resistant psychosis], and the meta-analyses perform quite well on the AMSTAR scale. Yet together, the meta-analyses published by this group of authors make up 6 out of the 12 meta-analyses on blood inflammatory/immunological markers in schizophrenia (20, 21, 28, 30, 31).

Based on these meta-analyses, alterations in cytokines, chemokines, lymphocytes, and oxidative stress markers have been demonstrated in the blood of patients with schizophrenia during an acute psychotic event which normalize with antipsychotic treatment (“state” markers IL1β, IL6, TGFβ and increased CD4/CD8 ratio), as opposed to other “trait” markers that remain elevated throughout the stages of the illness (20, 77). In general, effect sizes for FEP are similar in direction and magnitude to those in AR, indicating prior exposure to antipsychotic medication does not change the “acute” profile. Interestingly, a similar finding was reported in patients with chronic schizophrenia infected by *T. gondii*, with positive IgM antibody titers (a marker of acute/recent infection, or also potentially persistent infection or reinfection, possibly with a different genotype) linked to acute psychotic exacerbations (31). Thus it seems that differential patterns of immunological activation exist in different “states” of the schizophrenia illness course, in which patients who are experiencing an acute psychotic episode can be distinguished from those who are in symptomatic remission.

Yet the correlation between peripheral and brain inflammation is far from certain. Data from CSF samples might be better at representing brain immunity changes than the peripheral blood samples; however, such CSF samples are much less readily available (28). In their meta-analysis, Wang *et al*. identified 16 studies of cytokines in CSF, but did not stratify according to patients’ clinical state (28). Although the authors cautioned that their findings needed to be interpreted with caution due to the small numbers of studies and subjects, they reported that in schizophrenia many CSF alterations were also concordant with those reported in the peripheral blood. Communication between the systemic immune system and the brain and its consequence on microglia is a critical poorly understood component of the inflammatory response to systemic disease (78). Systemic infections activate neural and humoral pathways that communicate with the brain and initiate a coordinated set of metabolic and behavioral changes (79). However, these adaptive responses may become maladaptive when microglia have been “primed” by an ongoing pathology and respond to a systemic inflammatory challenge by switching their phenotype to an aggressive pro-inflammatory state (47), adversely affecting neuronal function and potentially leading to a psychotic decompensation through modulating effects of pro-inflammatory cytokines on neurotransmitter function (47).

Another recurring problem in the abovementioned clinical observational studies seems to be that although the effect sizes found in meta-analyses are moderate to large, for a majority of cytokines (or cytokine receptors or antagonists) assessed, there was significant heterogeneity in effect size estimates (20). Indeed it appears that due to the heterogeneity encountered, no individual assay seems apt to reliably differentiate between different patient groups in clinical practice, and large sample sizes are needed to detect these immunological disturbances which may reflect that they are either very low-grade and subtle differences, or they only occur in a subgroup of patients. The result is that both selection bias and methodological factors such as the choice of assay or analysis method and controlling for various confounding variables have a large impact on the results. In the meta-analysis on blood cytokine levels by Miller *et al*., 97% of studies controlled for age and gender as confounding factors, whereas the potential effects of race (41%), body mass index (35%), and smoking (24%) were often not considered (20). Furthermore, similarities have been found in the pattern of cytokine alterations during acute and chronic phases of illness in schizophrenia, bipolar disorder and major depressive disorder, pointing out the possibility of common underlying pathways that are not specific to schizophrenia (26).

## Clinical Trials of Immunomodulatory Drugs as Add-on Treatment for Schizophrenia

Following the concept that neuroinflammation may play a central role in the symptomatology and prognosis of schizophrenia, novel therapeutic prospects have arisen which aim to modulate immune effects to influence schizophrenia disease course. Non-steroidal anti-inflammatory drugs (NSAIDs) such as aspirin and celecoxib have been investigated for therapeutic intervention, as well as other compounds with anti-inflammatory properties which readily cross the blood–brain barrier such as davunetide (derived from activity-dependent neurotrophic protein (ANAP), a growth factor released by glial cells), fatty acids such as eicosapentaenoic acids and docosahexaenoic acids, estrogens, minocycline (a broad-spectrum tetracycline antibiotic with inhibitory effects on microglia), *N*-acetylcysteine (NAC), and even potent immunosuppressant drugs such as methotrexate. These drugs are being investigated as augmentation to antipsychotics (AP) in patients with a diagnosis of a schizophrenia spectrum disorders, and effects measured as change in symptom severity on the Positive and Negative Syndrome Scale (PANSS) or Brief Psychiatric Rating Scale (BPRS). The results of the most well-studied compounds (i.e., minocycline and NSAID) are summarized in Table 3. Well-designed clinical trials offer the clinical relevance the health-care field and community are waiting for. Since these trials are run with immunomodulatory drugs that are already available on the market, they come with the additional advantage of immediate usability and well-known clinical profiles in the case of positive outcome.

**Table 3:** Outcomes of meta-analyses of randomized controlled trials (RCTs) on minocycline and NSAID.

	*N* RCTs	Subjects	Treatment duration (weeks)	Total symptoms (PANSS total score or BPRS)	Positive symptoms (PANSS positive subscale or BPRS)	Negative symptoms (PANSS negative subscale or SANS)	General symptoms (PANSS general subscale)
**Minocycline**							
Sommer et al. (minocycline) (38)	4	182 drug–166 placebo	36 ± 18.8	SMD 0.22			
Oya et al. (minocycline) (39)	4	173 drug–157 placebo	25 ± 19.1	SMD 0.70*	SMD 0.26	SMD 0.86**	SMD 0.50*
Solmi et al. (minocycline) (40)	6	215 drug–198 placebo	19.7 ± 17.0	SMD 0.59*	SMD 0.22	SMD 0.76** (PANSS); SMD 0.60** (SANS)	SMD 0.44*
Xiang et al. (minocycline) (41)	8	286 drug–262 placebo	18.5 ± 13.4	SMD 0.64**	SMD 0.22*	SMD 0.69**	SMD 0.45*
**NSAID**							
Sommer et al. (NSAID) (36)	5	*N* = 264					
Nitta et al. (aspirin) (37)	2	133 drug–137 placebo	14 ± 2.8	Hedges *g* 0.29*			
Nitta et al. (celecoxib) (37)	6	255 drug–245 placebo	7.7 ± 2.1	Hedges *g* 0.21			
Sommer et al. (aspirin) (38)	2	133 drug–137 placebo	14 ± 2.8	Hedges *g* 0.30**			
Sommer et al. (celecoxib) (38)	5	236 drug–226 placebo	7.2 ± 2.4	Hedges *g* 0.15			
Zheng et al. (celecoxib) (42)	8	316 drug–310 placebo	8.3 ± 2.3	SMD 0.47**	SMD: 0.50**	SMD 0.32	SMD 0.35*

*SMD, standardized mean difference; PANSS, Positive and Negative Syndrome Scale; SMD, standard mean deviation; BPRS, Brief Psychiatric Rating Scale*.

*Sig at p = 0.05.

**Sig at p = 0.01.

A first meta-analysis on the effect of NSAIDs as add-on to AP was published by Sommer *et al*. in 2012 and included 5 double-blind, randomized, placebo-controlled trials (4 studies on celecoxib and 1 on acetylsalicylic acid), reporting on 264 patients. The authors reported a mean effect size of 0.43, which was significant at *P* = 0.02 in favor of NSAIDs on total symptom severity (36). This paper was subsequently criticized by Nitta *et al*., who replicated the meta-analysis, adding three more unpublished studies to the analysis demonstrating an overall less convincing case (37). Nitta *et al*. conducted subanalyses based on treatment setting and disease phase, demonstrating a significant improvement in PANSS total scores with NSAIDs in studies of inpatients (Hedges’ *g* = 0.44, *P* = 0.029) and first-episode patients (Hedges’ *g* = 0.39, *P* = 0.048), but not in outpatients and chronic patients (37).

In 2014, Sommer *et al*. reviewed double-blind randomized placebo-controlled trials of a broad range of immunomodulatory compounds. Weak to moderate beneficial effects were reported with the use of aspirin (Hedges’ *g* = 0.3, *P* = 0.001), NAC (Hedges’ *g* = 0.45, *P* = 0.009), and estrogens (Hedges’ *g* = 0.9, *P* = 0.001); while addition of celecoxib, EPA/DHA fatty acids, davunetide, and minocycline did not show efficacy (37, 38). That same year, a second meta-analysis by Oya *et al*. was published reviewing RCTs with minocycline (39). Compared to Sommer *et al*., this meta-analysis included two more original studies and excluded one study which was only published as congress proceeding. In this meta-analysis, minocycline was superior to placebo for decreasing PANSS total scores (39). Finally, during the course of 2017, two more meta-analyses on RCTs of minocycline in schizophrenia were published around the same time. Both Solmi *et al*. and Xhiang *et al*. demonstrated minocycline’s superiority versus placebo for reducing PANSS total scores, negative symptom scores, and general psychopathology scores. While SMDs for the different outcome measures are quite similar across the last three meta-analyses, only in Xiang *et al*. the effect on positive symptom scores reached significance level—probably due to the higher number of RCTs and study subjects included in this meta-analysis (40, 41). A third meta-analysis published in 2017 evaluated celecoxib as add-on for schizophrenia once more, this time demonstrating a significant improvement of total and positive symptoms scores (42).

Clearly, the major limitation of these studies is that for each compound, the number of individual studies is relatively low. As a result, even though they have been well executed and gain the highest scores on the AMSTAR assessment, the outcomes within and between the different meta-analyses remain relatively heterogeneous. It seems therefore too early to make conclusions on the efficacy on symptom severity of schizophrenia of augmentation with anti-inflammatory agents. Stratifying results per symptom domain makes sense, as celecoxib seems to be more effective against positive symptoms, and minocycline against negative symptoms. Overall, we would argue that too little information is currently available about the clinical determinants of groups that may benefit from these treatments to optimize study cohort selection, as well as the nature of the immune alterations—causing the choice of study drug to be virtually unguided and ranging a complete spectrum from food supplements to potent immunosuppressant drugs.

## The Microglial Phenotype

Besides the specific limitations of the studies mentioned above, one of the main problems underlying their limited clinical usefulness is our very limited basic knowledge about one of its key players in human brain, the microglial cell. Our understanding about the physiology of microglia is derived mainly from studies of tissue macrophages (43). However, in contrast to the latter, microglia are derived from myeloid precursors migrating from the yolk sac to the CNS early in embryonic development (E8.5) (80), and throughout life reside behind the blood–brain barrier where they are difficult to study *in vivo*. They account for 0.5–16.5% of the total number of cells in the human brain depending of the region explored (81). Microglia are highly plastic cells (73) that can adapt their behavior and morphology to changes in their environment and adopt different profiles (43). This defines microglial activation as a spectrum, in which transient microglial activation can include adaptive and beneficial physiological and behavioral responses, whereas maladaptive immune responses can lead to neuronal dysfunction and tissue damage. Microglia are critically involved in the organization of the neuronal network (44). They have an important role in synaptic pruning (51, 82) during brain development as well as in adult life, optimizing synaptic communication (83). Microglia can develop a range of functional phenotypes beyond the classic M1 (classical activation; an activation state in which microglia would adopt a deleterious function) versus M2 (alternative activation; in which immune cells adopt a regulatory or tolerance-inducing profile) paradigm (43). However, it is the specific manner in which microglia are activated and the phenotype that they adopt that is important in determining the influence of microglia in neurodegeneration (78). Morphology does not indicate the function of microglia, which means that even if the presence of enhanced microglial activation in certain patient groups or disease states is proven unequivocally, we still do not know what this means in terms of microglial activity and whether to interpret this as beneficial or harmful. The activation may be pathological or part of an endogenous compensatory response to some other aspect of the disease process.

Neuropathological studies remain the gold standard to gain answers to these questions. Therefore, the use of a single microglial marker, often different between the *post-mortem* studies (Iba1, CD68, or HLA-DR), is not sufficient to identify the phenotype expressed by microglia. Furthermore, if their phenotype is related to a certain clinical state or illness phase, knowledge of the time course of the microglial activation is relevant. Adding to the complexity of microglia, some authors have argued that the microglial activation status is most probably a reflection of the history of life events [defined as innate immune memory (84)], including prenatal and *perimortem* influences, as well as an individual’s genetic background (85), emphasizing the need for detailed clinical information in *post-mortem* or PET investigations of microglia.

## Neuroplasticity and Microglia

Neuroplasticity refers to the ability of the brain to develop and finetune its neural connections, including adjustments in response to changes in the environment, by alteration in the neurons or glial cells *via* cell division/apoptosis and synaptic/neurite remodeling. Microglia are key facilitators for neuronal plasticity during brain development (44, 45) and in adult life (86). Constant surveillance of the microenvironment by microglial processes (73) and their attraction to active rather than non-stimulated synapses imply that microglia might monitor the functional state of synapses leading to plastic changes in healthy adult brain (87). This could occur through remodeling of the extracellular spaces and elimination of synaptic elements, regulation of the neurotransmitters present in the synaptic cleft such as glutamate, or by direct contact with synaptic elements (87, 88) *via* the complement components (51).

Research, summarized in this paper, has identified alterations in the key actors and mechanisms involved in neuroplasticity. A quantitative meta-analytic summary of studies focused on neuron density provides support for the finding of altered neuron density in schizophrenia, with variation dependent on age (14). The vertical micro-circuits within the cortex, known as minicolumns, normally become thinner with age in controls (89), indicating a reduction in neuropil, but not in schizophrenia (90). The role that microglia may play in this process is strongly suggested by the finding that microglia are involved in synaptic pruning during healthy brain development (28). Given that neuropil expansion is a correlate of synapse number, an abnormality of microglia may be understood to be a basis for reduced synaptic and neuropil modulation.

The quantitative neuropathological changes in the cerebral cortex of schizophrenia, suggest that the “reduced neuropil hypothesis” (91) applies in early life in schizophrenia, but subsequently it is “reduced neuroplasticity” which leaves the cells and minicolumns relatively widely spaced in later life, compared to the typical pattern of healthy control neuropil thinning. This is consistent with neuroimaging data and offers a microanatomical explanation to account for many of the larger scale functional–anatomical changes observed in structural neuroimaging studies (14), which have shown that over time schizophrenia is associated with progressive decrease of whole brain volume and whole brain gray matter (92), frontal gray and white matter, parietal white matter, and temporal white matter (93).

Consequently, an altered aging trajectory is implicated as a factor in the pathogenesis of schizophrenia. This hypothesis is supported by the finding that the decrease in brain-derived neurotrophic factor (BDNF) which occurs in older age is amplified in schizophrenia. BDNF is a regulator of microglial-mediated synaptic plasticity (94), suggesting a greater reduction in neuroplasticity with advancing age in schizophrenia (95). The progressive anatomical abnormalities of brain structure recorded in longitudinal studies indicating greater severity following the first episode of illness may be the cumulative effect of reduced neuroplasticity over time. One of the consequences at the level of symptoms may be that a shift from positive symptoms to more negative symptoms, which has been reported during the disease course, is consistent with the implications of reduced plasticity—i.e., negative symptoms represent a relative loss of functions, that may become more dominant due to insufficient plasticity to keep up with ongoing cognitive demand, rather than the presence of additional cognitive phenomena described as positive symptoms.

## Conclusion and Future Directions: Multimodality as Key to the Future of Neuroinflammation Research in Schizophrenia

The evidence from the psychoneuroimmunology research field converges on a progressive illness model of schizophrenia in which primed microglia contribute to altered neuroplasticity, leading to structural and chemical abnormalities that accumulate as patients age. This model integrates both neurodevelopmental and neurodegenerative hypotheses in schizophrenia, as the combination of a pre- or perinatal immunological vulnerability serves as a background for ongoing abnormal peripheral and brain immune responses which throughout life constitute a basis for illness exacerbation and progressive defects. Yet although the psychoneuroimmunology field provides a compelling pathophysiological model, at this moment it has not attained a clinically significant level of relevance to comprehensively explain the etiology, diagnosis or potential treatment of the disorder. In addition, many of the findings appear non-specific to schizophrenia. To enhance the clinical relevance of this field, further links are needed to connect the immune-related hypotheses to other major research fields and hypotheses of schizophrenia psychopathology.

Such an interesting new link between immune alterations and neuroplasticity, in which aberrant microglial activation could cause reduced synaptic and neuropil modulation, has recently emerged, but still needs to be comprehensively investigated in the human brain. Furthermore, knowledge on the physiology and functional phenotypes of microglia, as well as their link with systemic inflammation, is crucially insufficient to interpret current findings. We would therefore argue that studies acting as a bridge between the preclinical and clinical studies are crucially missing. We therefore advocate multimodal research studies that explore neuroinflammatory mechanisms both in experimental models and schizophrenia patients; using *in vivo* multimodal imaging and analysis of immunological markers and microarchitectural changes in *post-mortem* brain tissue as translational means. In particular, the underutilized human brain approach has a powerful translational nature in complex neuropsychiatric conditions (9). Overcoming individual limitations, this combined approach evidently generates added value and has a high feasibility, as it is supported by literature, preliminary data, and active involvement of highly skilled research teams situated within the field that already possess the necessary know-how. The advent of a greater recognition of the role of the immune system and its effects on neuroplasticity in schizophrenia will allow identification of potential novel therapeutic avenues, thereby finally bringing the psychoneuroimmunology research field to its full potential.

## Author Contributions

All authors met ICMJE criteria and all those who fulfilled those criteria were listed as authors. All authors had access to the study data and made the final decision about where to present these data.

## Conflict of Interest Statement

The authors declare that the research was conducted in the absence of any commercial or financial relationships that could be construed as a potential conflict of interest.

## Abbreviations

BPRS, Brief Psychiatric Rating Scale; CNS, central nervous system; CSF, cerebrospinal fluid; FEP, first-episode psychosis; GWAS, genome-wide association study; IL, interleukin; MIA, maternal immune activation; MA, meta-analysis; MRI, magnetic resonance imaging; PANSS, Positive and Negative Syndrome Scale; NAC, *N*-acetylcysteine; PET, positron emission tomography; SAPS, Scale for the Assessment of Positive Symptoms; SR, systematic review; TSPO, translocator protein.
